# STFTransNet: A Transformer Based Spatial Temporal Fusion Network for Enhanced Multimodal Driver Inattention State Recognition System

**DOI:** 10.3390/s25185819

**Published:** 2025-09-18

**Authors:** Minjun Kim, Gyuho Choi

**Affiliations:** Department of Artificial Intelligence Engineering, Chosun University, 309, Pilmun-daero, Dong-gu, Gwangju 61452, Republic of Korea; 20223379@chosun.ac.kr

**Keywords:** STFTransNet, transformer based spatial temporal fusion, driver inattention state recognition, partial occlusion, multimodal drowsiness/distraction detection

## Abstract

Recently, studies on driver inattention state recognition as an advanced mobility application technology are being actively conducted to prevent traffic accidents caused by driver drowsiness and distraction. The driver inattention state recognition system is a technology that recognizes drowsiness and distraction by using driver behavior, biosignals, and vehicle data characteristics. Existing driver drowsiness detection systems are wearable accessories that have partial occlusion of facial features and light scattering due to changes in internal and external lighting, which results in momentary image resolution degradation, making it difficult to recognize the driver’s condition. In this paper, we propose a transformer based spatial temporal fusion network (STFTransNet) that fuses multi-modality information for improved driver inattention state recognition in images where the driver’s face is partially occluded by wearing accessories and the instantaneous resolution is degraded due to light scattering from changes in lighting in a driving environment. The proposed STFTransNet consists of (i) a mediapipe face mesh-based facial landmark extraction process for facial feature extraction, (ii) an RCN-based two-stream cross-attention process for learning spatial features of driver face and body action images, (iii) a TCN-based temporal feature extraction process for learning temporal features of extracted features, and (iv) an ensemble of spatial and temporal features and a classification process to recognize the final driver state. As a result of the experiment, the proposed STFTransNet achieved an accuracy of 4.56% better than the existing VBFLLFA model in the NTHU-DDD public DB, 3.48% better than the existing InceptionV3 + HRNN model in the StateFarm public DB, and 3.78% better than the existing VBFLLFA model in the YawDD public DB. The proposed STFTransNet is designed as a two-stream network that can input the driver’s face and action images and solves the degradation in driver inattention state recognition performance due to partial facial feature occlusion and light blur through spatial feature and temporal feature fusion.

## 1. Introduction

Recently, studies on driver inattention state recognition as an advanced mobility application technology are being actively conducted to prevent traffic accidents caused by driver drowsiness and distraction. Drowsiness and distraction during driving reduce the driver’s ability to understand road conditions and increase the risk of traffic accidents. The National Highway Traffic Safety Administration (NHTSA) in the United States reported that approximately 1500 deaths were due to drowsy driving accidents and approximately 3308 deaths were due to driver distraction accidents in 2022 [1,2]. The main causes of drowsy driving are a lack of sleep, long driving hours, and drinking, and the main causes of distraction are the use of electronic devices, conversations with passengers, and eating. The Foundation for Traffic Safety (FTS) in the United States reported that the rate of driver speeding increased by up to 19% and the rate of drowsy driving increased by up to 5.4% due to an increased reliance on advanced driver assistance systems (ADAS) developed for driver convenience [3]. The risk of accidents increases when drowsiness and distraction occur together. Accordingly, advanced countries such as the US and Europe are researching and developing to apply drowsiness recognition and distraction recognition technologies to ADAS.

Driver inattention state recognition systems are typically divided into driver drowsiness recognition systems and driver distraction recognition systems [4]. Driver drowsiness recognition systems are being studied using vehicle operation data, driver driving behavior characteristics, and biosignals, while driver distraction recognition systems are being studied using driver driving behavior characteristics. Singh et al. [5] developed a system that recognizes drowsiness using the variability of resistance according to the strength of steering wheel grip to recognize driver drowsiness. State recognition systems using vehicle operation data have low accuracy due to external variables such as weather, road conditions, and traffic conditions. Chaabene et al. [6] developed a drowsiness recognition system using a convolutional neural network (CNN) model to recognize driver drowsiness using EEG signals acquired from 14 channels with an electroencephalogram (EEG) measurement device, Emotiv EPOC. State recognition systems using driver biosignals interfere with driving due to the wearing of biosignal acquisition equipment while driving. The state recognition system using behavioral features is being actively studied because it can recognize the state without interference with driving by using a camera installed inside the vehicle, rather than the driver’s equipment while driving.

Zandi et al. [7] developed a drowsiness detection system using driver’s eye tracking data using random forest (RF) and support vector machine (SVM) to recognize driver’s drowsiness. The RF and SVM-based drowsiness detection system was analyzed to have low drowsiness recognition accuracy by using features that did not include location information in the image. Tamanani et al. [8] developed a drowsiness recognition system based on the driver’s facial features using a CNN to recognize driver drowsiness. The CNN-based drowsiness recognition system improved the drowsiness recognition accuracy by using features that include location information in the image through convolution operations, compared to machine learning. Deng et al. [9] developed a CNN-based DriCare drowsiness recognition system using the driver’s eye and mouth features to recognize the driver’s drowsiness. The DriCare-based drowsiness recognition system analyzed whether the eyes were open and measured the ratio of the height and width of the mouth to improve yawning and drowsiness recognition accuracy. Huang et al. [10] developed a DenseNet-based alternative wide group residual densely (AWGRD) driver inattention state recognition system using driver driving behavior features for driver inattention state recognition. The AWGRD system improved driver inattention state recognition performance by using a model that combines the DenseNet structure and residual network. Existing driver inattention state recognition systems extract features centered on location information using machine learning and CNN models. Location-based driver inattention state recognition has poor state recognition accuracy due to partially occluded images of the face caused by accessories and resolution degradation caused by lighting changes.

In this paper, we propose a transformer based spatial temporal fusion network (STFTransNet) that fuses multi-modality information for improved driver inattention state recognition in images of partially occluded facial features caused by accessories while driving and images with reduced resolution due to light scattering caused by lighting changes. The proposed STFTransNet consists of (i) a mediapipe face mesh-based facial landmark extraction process for facial feature extraction, (ii) an RCN-based two-stream cross-attention process for learning spatial features of driver face and body action images, (iii) a TCN-based temporal feature extraction process for learning temporal features between extracted features, and (iv) an ensemble of spatial and temporal features and a classification process to recognize the final driver state. As a result of the experiment, the proposed STFTransNet model achieved an accuracy of 4.56% better than the existing VBFLLFA [11] model in the National Tsing Hua University Drowsy Driver Detection (NTHU-DDD) public DB, an accuracy of 3.48% better than the existing InceptionV3 + HRNN [12] model in the StateFarm public DB, and an accuracy of 3.78% better than the existing VBFLLFA [11] model in the YawDD public DB. The proposed STFTransNet is designed as a two-stream network that can input the driver’s face and action images and solves the degradation of driver inattention state recognition performance due to partial facial feature occlusion and light blur through spatial feature and temporal feature fusion. In addition, STFTransNet contributes to the development of an improved driver inattention state recognition system by additionally recognizing the driver’s distraction state as well as drowsy state.

This paper is structured as follows: Section 1 introduces the background, motivation, and objectives of this study. Section 2 reviews the related works, providing a comprehensive overview of existing approaches and the limitations of driver state inattention detection system studies. Section 3 describes the proposed model’s architecture and methodology in detail, including its key components and innovations. Section 4 presents the results of both comparative experiments and our self-conducted experiments, followed by an in-depth analysis of these findings. Finally, Section 5 concludes the paper with a summary of the study, highlighting the key contributions and offering insights into future research directions.

## 2. Materials and Methods

**Figure 1:**
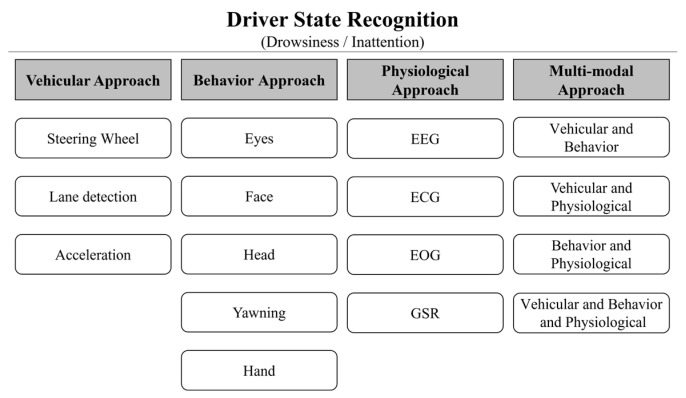
Classification structure of driver inattention state recognition system.

Driver inattention state recognition systems are categorized based on the type of input data, as shown in Figure 1. Driver inattention state recognition systems are divided into driver drowsiness detection (DDD) and driver inattention detection (DID). DDD and DID systems utilize vehicle operation characteristics, behavioral characteristics, and biosignal characteristics that can be acquired from the driver while driving.

The state recognition system, using the driver’s vehicle operation data during driving, analyzes the vehicle driving pattern from the steering wheel movement, braking pattern, and lane departure measurement and recognizes the driver’s drowsiness and distraction. The state recognition system, using the driver’s behavioral characteristics during driving, recognizes the driver’s drowsiness and distraction using the driver’s gaze, eye, mouth, head movement, and body posture change characteristics. The state recognition system using the driver’s biosignals during driving recognizes the driver’s drowsiness and distraction by analyzing the driver’s electrocardiogram (ECG), electromyogram (EMG), EEG, and respiration. Recently, a multimodal-based driver inattention state recognition system has been studied using the driver’s vehicle operation information data, driving behavioral characteristics, and biosignals, which are fused with two or more 1D signals and 2D image data [13,14,15]. Table 1 provides information on a comparative analysis of existing driver inattention detection technologies, organized by data type, acquisition, dataset, network, detection state, and accuracy.

### 2.1. Drowsiness Detection System Using Vehicle Operation Feature

Mcdonald et al. [16] developed a drowsiness recognition system that detects lane departure in real time by analyzing the wheel angle due to driver drowsiness using RF. The RF-based drowsiness recognition system found that drivers with a large variability in vehicle speed had a higher correlation with drowsiness than those with a small variability in speed. The RF-based drowsiness recognition system was verified to achieve 79% drowsiness recognition accuracy using the National Advanced Driving Simulator public DB. Dehzangi et al. [29] developed a drowsiness recognition system using data that analyzed the acceleration, braking, and steering wheel axis patterns of the vehicle using a decision tree (DT). The DT-based drowsiness recognition system recognizes by using vehicle operation features acquired by the driver for 4.4 s while driving. The DT-based drowsiness recognition system was verified to achieve 99.1% drowsiness recognition accuracy based on the Karolinska sleepiness scale (KSS) index criteria, in which subjects directly indicated the degree of drowsiness using their own acquired DB. Arefnezhad et al. [17] developed a driver drowsiness recognition system using an adaptive neuro-fuzzy inference system (ANFIS) based on a neuro-fuzzy system using steering wheel axis features. The ANFIS-based drowsiness recognition system was verified to achieve a drowsiness recognition accuracy of 98.12% and an AUC of 97% using the BI301Semi public DB of Khajeh Nasir Toosi University of Technology. The state recognition system using vehicle operation features while driving has limitations of low accuracy due to weather, road conditions, and traffic conditions.

### 2.2. Drowsiness Detection System Using Driver’s Driving Behavior Feature

A state recognition system using the driver’s driving behavior characteristics is being studied using computer vision technology after capturing the driver’s appearance with a camera. Liu et al. [30] developed a drowsiness recognition system that extracts features in spatial and temporal dimensions using a 3DCNN from the behavior characteristics of urban railway drivers. 3DCNN was verified to achieve 98.41% accuracy in drowsiness recognition using the KTH public DB. State recognition research using computer vision technology is being conducted not only on driver inattention state recognition in transportation, but also on diseases. Cruz et al. [31] developed an eye recognition system to prevent computer vision syndrome using a long-term recurrent convolutional network (LRCN). The LRCN-based eye recognition system was verified to have a 97.9% F1-score for eye blink recognition using the Talking Face public DB and a 91% F1-score for eye state recognition using EyeBlink8 public DB. State recognition systems based on driver behavior are being actively studied as they develop from traditional statistical techniques and machine learning techniques to deep learning techniques due to the development of AI technology. Ghourabi et al. [18] developed a drowsiness recognition system using eye aspect ratio (EAR) and mouth aspect ratio (MAR) using a multi-perceptron and K-NN. EAR and MAR are indicators of the degree of opening of the eyes and mouth and are used to recognize eye blinks, yawns, etc. The multi-perceptron and K-NN-based drowsiness recognition system was verified to achieve 94.31% yawn recognition accuracy and 71.74% eye blink recognition accuracy using the NTHU-DDD public DB. Ahmed et al. [32] developed a driver drowsiness recognition system using eye and mouth images using CNN and VGG16 models. The CNN and VGG16-based drowsiness recognition system was verified to achieve 97% drowsiness recognition accuracy in the CNN model and 74% drowsiness recognition accuracy in the VGG-16 model using a DB that classified 2900 self-acquired images into four categories (open eyes, close eyes, yawn, and non-yawn). Kayadibi et al. [33] developed a deep convolutional neural network (DCNN)-based drowsiness recognition system using AlexNet. The DCNN system was verified to achieve an eye state recognition accuracy of 97.32%, an AUC of 99.37%, and an F1-score of 94.67% using the ZJU public DB, and with an eye state recognition accuracy of 97.93%, an AUC of 99.69%, and an F1-score of 97.92% using the CEW public DB. Research using deep learning techniques for driver behavior-based systems is actively being conducted using the attention technique and the transformer model to solve the limitations of CNN, which loses sequential information, and LSTM, which has limited parallel processing. Yang et al. [11] developed a driver drowsiness detection system based on face images by designing a two-branch multi-head attention (TB-MHA) module and extracting temporal and spatial information features. The TB-MHA system analyzed face movement and eye and mouth movement information using facial landmarks and local face regions. The TB-MHA system was verified to achieve 95.2% drowsiness recognition accuracy using the YawDD public DB, 91.3% drowsiness recognition accuracy using the NTHU-DDD public DB, and 97.8% drowsiness recognition accuracy using the VBDDD self-acquired DB. Xiao et al. [19] developed a driver fatigue recognition system based on facial feature points by designing a fatigue driving recognition method based on feature parameter images and a residual swin transformer (FPIRST). The FPIRST system generates parameter images based on facial feature points and recognizes driver fatigue state through a residual swin transformer network. The FPIRST system was verified to achieve 96.51% driver fatigue recognition accuracy using the HNUFD public DB. Huang et al. [20] developed a driver fatigue detection system based on driver facial images by designing a self-supervised multi-granularity graph attention network (SMGA-Net). The SMGA-Net system optimized the hyperparameters of the network by transforming and then restoring the original image. The SMGA-Net system recognizes driver fatigue by combining spatial features extracted using VGG-16 and temporal features extracted by BiLSTM designed with graph attention. The SMGA-Net system was verified to achieve 81% accuracy and an 81.13% F1-score of driver fatigue recognition using the NTHU-DDD public DB. Xu et al. [34] proposed a driver drowsiness detection system that combines an improved YOLOv5s with a lightweight backbone and DeepSort tracking to improve frame-by-frame drowsiness detection accuracy and continuous tracking stability. The proposed improved YOLOv5s + DeepSort system uses a MobileNet_ECA lightweight backbone and a triplet attention module (TAM) neck and combines DeepSort-based PERCLO, continuous eye closure, and continuous yawn frame counts to compensate for persistent detection failures and information loss issues. The improved YOLOv5s + DeepSort system was verified to achieve a driver drowsiness detection accuracy of 97.4% using the YAWDD public database.

A study on a system that recognizes driver drowsiness as well as driver distraction while driving is in progress. Huang et al. [10] designed an alternative wide group residual densely (AWGRD) based on the DenseNet structure and developed an abnormal driving behavior recognition system using driver driving behavior images. The AWGRD system was verified to achieve 95.97% accuracy and a 96% F1-score of abnormal driving behavior detection based on 10 driving patterns using the StateFarm public DB. Alotaibi et al. [12] designed an ensemble deep learning model combining ResNet, Inception module, and HRNN and developed a driver distraction recognition system using driver driving behavior images. The ensemble deep learning system was verified to achieve 99.30% accuracy for driver distraction recognition using the StateFarm public DB and 92.36% accuracy for driver distraction recognition using the AUC public DB. Tran et al. [35] developed a system to recognize driver distraction based on driver behavioral features using VGG-16, AlexNet, GoogleNet, and residual networks. The transfer learning-based distraction recognition system was verified to achieve 86% accuracy for VGG-16, 89% accuracy for AlexNet, 89% accuracy for GoogleNet, and 92% accuracy for ResNet using a self-acquired DB acquired based on 10 distraction behaviors. The authors argued that although the accuracy of GoogleNet is lower than that of ResNet, GoogleNet is more suitable for real-time state recognition, considering the processing speed, which is 11 Hz for GoogleNet and 8 Hz for ResNet. The driver inattention state recognition system based on driver driving behavior has limitations due to occlusion by accessories worn on the face and resolution degradation due to lighting changes.

### 2.3. Drowsiness Detection System Using Biosignals

The study of a driver inattention state recognition system based on biosignals is in progress, utilizing ECG, PPG, and EEG biosignal data. Gangadharan et al. [21] developed a drowsiness recognition system using EEG signals via SVM machine learning. The SVM-based drowsiness recognition system acquired EEG data from 18 subjects wearing Muse-2 EEG headband while taking a nap and recognized drowsiness using AR first-order coefficient, AR second-order coefficient, and LRSSV features derived from temporal electrodes. The SVM-based system was verified to achieve 78.3% drowsiness recognition accuracy using self-acquired EEG DB. Shahbakhti et al. [22] designed a VME-PCA-DWT system to develop a drowsiness recognition system using eye-blink detection and removal filtering from EEG data. The VME-PCA-DWT system was verified to achieve 93% accuracy in drowsiness recognition using self-acquisition DB1 [23], 92% accuracy in drowsiness recognition using self-acquisition DB2 [24], and 71.1% accuracy in drowsiness recognition using self-acquisition DB3 [25]. Chaabene et al. [6] developed a drowsiness recognition system using EEG data acquired by an Emotiv EPOC + headset using a CNN network. The CNN network-based drowsiness recognition system was verified to achieve 97.8% accuracy in drowsiness detection using a self-acquisition DB. The biosignal-based system has limitations in that it reduces the driver’s concentration due to wearing biosignal acquisition equipment that interferes with driving.

### 2.4. Multimodal-Based Drowsiness Detection System

Recently, studies on driver inattention state recognition systems using multidimensional features by integrating vehicle, driver behavior, and driver biosignal data are being conducted. Arefnezhad et al. [26] developed a multimodal driver drowsiness recognition system by integrating data of lateral deviation and acceleration, steering wheel angle data, and ECG signals using KNN and RF models. The KNN and RF model-based drowsiness recognition system analyzed the possibility of improving drowsiness recognition accuracy by integrating data through multidimensional analysis. The KNN and RF model-based drowsiness recognition system was verified to achieve a drowsiness recognition accuracy of 91.2% using self-acquired vehicle DB and ECG DB. Abbas et al. [27] designed HybridFatigue and developed a multimodal driver drowsiness recognition system by combining PERCLOS and ECG. The HybridFatigue system was verified to achieve a drowsiness recognition accuracy of 94.5% by combining PERCLOS and ECG after pre-training with 4250 images from CAVE-DB, DROZY, and CEW public DBs. Gwak et al. [28] developed a multimodal drowsiness recognition system that can recognize shallow drowsiness states by combining vehicle, driver behavior, and driver biosignals using an ensemble model and an RF model that combined linear regression (LR), SVM, and KNN. The multimodal drowsiness recognition system was tested using self-acquired steering wheel DB, driver eye feature DB, EEG DB, and ECG DB. The ensemble model and RF model were verified to have an accuracy of 82.4% for recognizing alert and slightly drowsy states and 95.4% for recognizing alert and moderately drowsy states by merging self-acquired DBs.

Existing driver drowsiness detection systems were designed based on driving operation data, driver behavior characteristics, biosignals, and multimodal data. Drowsiness detection systems utilizing vehicle operation data, driver behavior characteristics, and biosignals have limitations, including reduced state recognition accuracy due to external variables, partial face occlusion caused by accessories, and driving interference resulting from the wearing of biosignal measurement equipment. Multimodal-based drowsiness detection systems have difficulties in real-time processing and the complexity that occurs during the data fusion process. This study proposes STFTransNet, which uses multiple facial and body action features of the driver and enables improved driver inattention state recognition through the fusion of spatial and temporal features to overcome the limitations of image quality degradation due to partial occlusion of the face and light scattering.

## 3. Transformer Based Spatial Temporal Fusion Network

**Figure 2:**
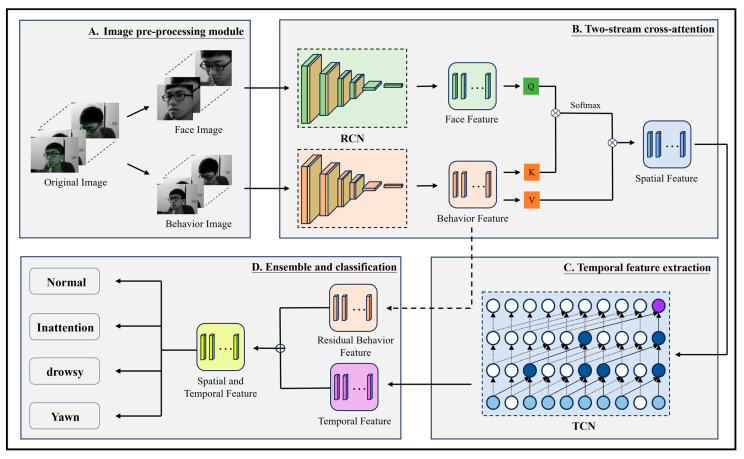
Structure of the proposed STFTransNet system.

In this paper, we propose a driver inattention state recognition system based on STFTransNet to solve the partial occlusion problem of the existing face and the limitations of image quality degradation due to light spillover, as shown in Figure 2. The proposed STFTransNet consists of the following processes: (i) mediapipe face mesh-based facial landmark extraction process to extract facial features, (ii) RCN-based two-stream cross-attention process to learn spatial features of driver face and body action images, (iii) a TCN-based temporal feature extraction process to learn temporal features between the extracted features, and (iv) an ensemble of spatial and temporal features and classification process to recognize the final driver state.

### 3.1. Mediapipe Face Mesh-Based Facial Landmark Extraction Process

**Figure 3:**
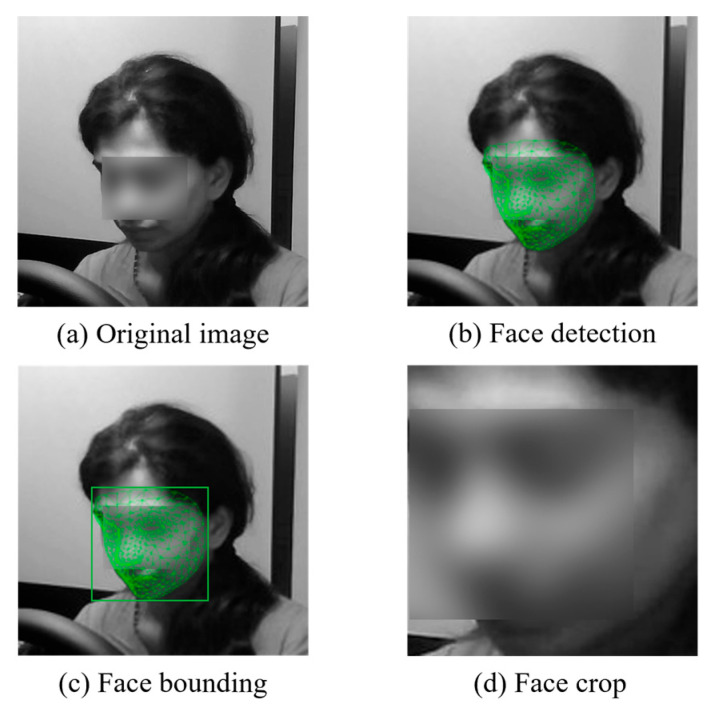
Face image preprocessing process using NTHU-DDD DB.

The mediapipe face mesh-based facial landmark extraction process simultaneously learns the driver’s physical behavior and face and extracts facial features to improve the failure of state recognition due to partial occlusion of the face. This paper uses the mediapipe face mesh to recognize faces in original images and extract facial features. The mediapipe face mesh is an open-source framework developed by Google, which provides 468 3D facial landmarks to create an accurate face model [36]. The mediapipe face mesh can extract facial landmarks in real time, making it suitable for driver state monitoring. Figure 3 shows the facial feature extraction process of the NTHU-DDD DB. Figure 3a shows the original image used for preprocessing, Figure 3b shows the process of recognizing faces using the mediapipe face mesh, Figure 3c shows the process of bounding to extract the face region, and Figure 3d shows the image from which the face region is extracted. The proposed STFTransNet uses a dual input path, unlike existing single-face inputs. The STFTransNet input image simultaneously contains the original frame Iorig and the cropped face Iface, allowing it to leverage driver behavior features contained in Iorig even when partially occluded.

### 3.2. RCN-Based Two-Stream Cross-Attention Process

**Figure 4:**
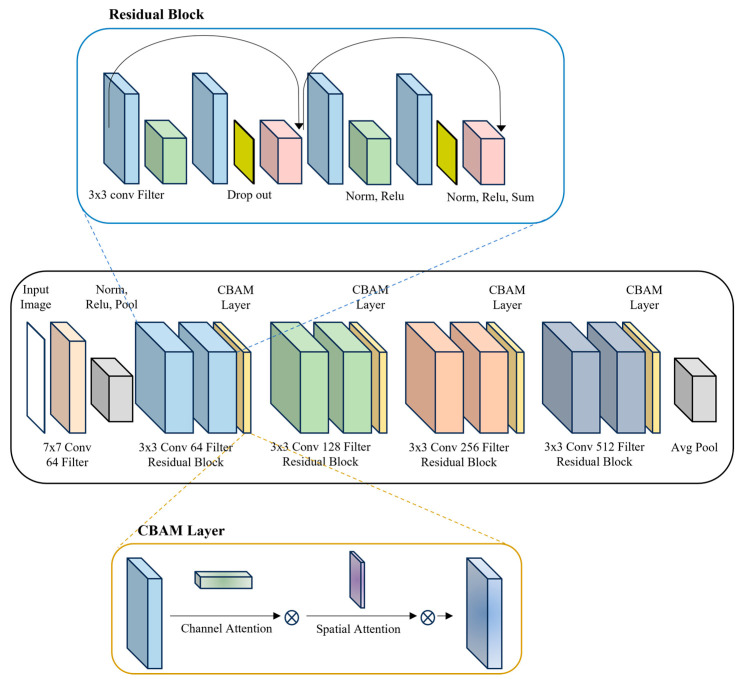
RCN structure diagram.

RCN uses ResNet18 combined with CBAM, consisting of channel attention and spatial attention. Figure 4 shows the architecture of the RCN network.

ResNet18 is a residual network consisting of four residual blocks and a skip connection structure that adds a portion of the input value to the output value of each block [37]. The operation through the skip connection is performed with both the input ***x*** and the function ***F(x),*** being four-dimensional tensors $T\in R^{\left(N\times C\times H\times W\right)}$. ***N*** is the batch size, ***C*** is the number of channels, ***H*** is the height, and ***W*** is the width. Equation (1) computes the skip connection structure.

(1) $$H\left(x\right)=x+F(x)$$

CBAM is a module that sequentially applies channel attention and spatial attention to emphasize important information in input features [38]. The channel attention layer of CBAM learns the importance of each channel of the input feature map and gives high weights to important channels. The channel attention layer extracts global information in the form of $R^{\left(N\times H\times W\right)}$ using average pooling and max pooling, respectively, to learn channel importance. The extracted features are input to the MLP to generate a feature map indicating the importance of the channel. The generated feature map is scaled to a value between 0 and 1 using the sigmoid function to generate a feature map ***M_C_(F)*** for each channel. Equation (2) calculates channel attention, and ***σ*** is the sigmoid function.

(2) $$M_{c}(F)=\sigma (MLP(AvgPool(F))+MLP(MaxPool(F))$$

Spatial attention learns the importance of each location in the input feature map and assigns high weights to important locations. To learn the importance of locations, spatial attention extracts spatial information of the size $R^{\left(N\times H\times W\right)}$ by using average pooling and max pooling along the channel dimension, respectively. The extracted features are combined in the channel direction and input to the convolution operation to generate a spatial feature map. The generated feature map scales the importance of each location to a value between 0 and 1 through the sigmoid function to generate ***M_S_(F)*** for each channel. Equation (3) calculates spatial attention.

(3) $$M_{s}(F)=\sigma (Conv([AvgPool(F);MaxPool(F)]))$$

RCN is a structure that applies CBAM to the output feature map generated from each residual block of ResNet18 to emphasize important spatial information and use it as the input of the next residual block. RCN uses the original image ***I_orig_*** and the face image ***I_face_*** as two-stream input images. ***I_orig_*** and ***I_face_*** pass through the residual block to generate ***F_orig_*** and ***F_face_***, and apply CBAM to generate ***F’_orig_*** and ***F’_face_***.

**Figure 5:**
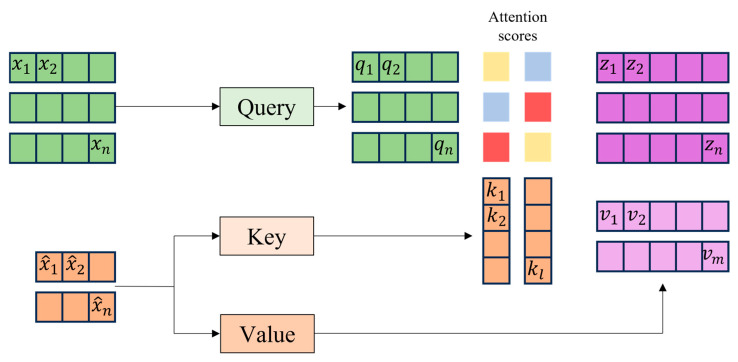
Cross-attention structure diagram.

The RCN-based two-stream cross-attention process is a structure applied to fuse ***F’_orig_*** and ***F’_face_***, as shown in Figure 5. Cross-attention emphasizes important information through the interaction between ***F’_orig_*** and ***F’_face_*** extracted using RCN [39]. The cross-attention module uses ***F’_face_*** as a query and ***F’_orig_*** as a key and value. The cross-attention module generates an attention map by performing a dot product to calculate the similarity between the query and key and normalizes it using the softmax function. The normalized attention map performs a dot product with the value to output a feature map ***F_O_*** that emphasizes important information between the original image and the face image. Equation (4) and Equation (5) compute cross-attention. The proposed STFTransNet does not simply concatenate the two existing inputs, but cross-attentions the original image and the face image to give weight to important information between the original and the face.

(4) $$A=softmax(Q\cdot K^{T})$$

(5) $$F_{O}=A\cdot V$$

### 3.3. TCN-Based Temporal Feature Extraction Process

**Figure 6:**
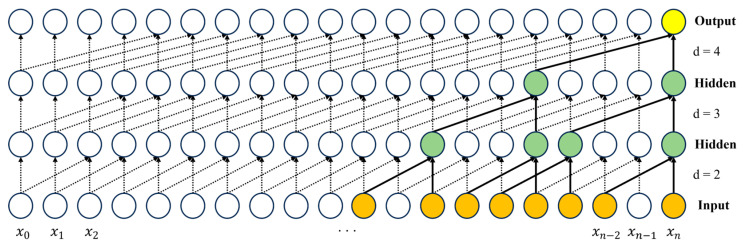
TCN structure diagram.

Figure 6 shows the structure of TCN used to learn the temporal information of images in the TCN-based temporal feature extraction process.

TCN is a neural network structure that can effectively learn the temporal order and long-term dependency of sequence data through a convolutional structure and an extended receptive field [40]. The feature ***F_O_*** extracted through cross-attention is used as input to TCN to extract temporal features in batch units. ***F_O_*** is converted to sequence form to be used as input to TCN. The dilated convolution of TCN extends the receptive field to learn a wider range of sequence information. Equation (6) represents the dilated convolution operation, which calculates the output ***y(t)*** using the input sequence ***x(t)*** and filter ***f(i)***. ***k*** represents the kernel size, and ***r*** represents the dilation rate. ***y(t)*** is generated as a result of combining multi-scale information of multiple time points in ***x(t)*** through dilated convolution.

(6) $$y\left(t\right)=\sum_{i=0}^{k-1}f(i)\cdot x(t-r\cdot i)$$

TCN alleviates the problem of vanishing gradients, where the gradients gradually decrease as the layers become deeper during the backpropagation process through residual connections. The TCN structure of STFTransNet consists of three layers and gradually expands the receptive field to learn complex sequence patterns. ***F_TI_***, which is converted into the input of TCN, is output as ***F_TO_*** after learning. The proposed STFTransNet is configured to learn temporal feature information aligned with spatial information while simultaneously extracting spatial features, unlike existing temporal feature followed by spatial feature extraction methods.

### 3.4. Ensemble of Spatial and Temporal Features and Classification Process

The ***F_TO_*** output by TCN is converted to the original batch form, and the residual ***F’_orig_*** output by RCN is added to output the final combined feature ***F_combined_***. The original image comprehensively includes the driver’s facial features and behavioral features, and it helps improve the driver status recognition performance by ensembling with ***F_TO_***. Equation (7) inputs the combined feature ***F_combined_*** to the fully connected layer and calculates the softmax function to predict the driver’s status. The proposed STFTransNet, unlike existing temporal feature post-spatial feature extraction methods, recognizes the final driver inattention state by concatenating the output of the temporal module and the residual spatial features of raw data to emphasize driver behavioral features in situations where facial information is insufficient.

(7) $$Prediction=Softmax(Linear\left(F_{combined}\right))$$

## 4. Experimental Studies

The experimental environment for evaluating the performance of driver inattention state recognition using the proposed STFTransNet in this paper is an Intel (R) Core i5-13600 K CPU, 32 GB of RAM, and NVIDIA RTX 4090 GPU for hardware and an Ubuntu 22.04 Visual Studio Code for software. The public DBs used to evaluate the performance of driver inattention state recognition using the proposed STFTransNet are NTHU-DDD, YawDD, and StateFarm. The NTHU-DDD DB consists of data acquired from 36 subjects ‘wearing glasses during the day’, ‘not wearing glasses during the day’, ‘wearing sunglasses during the day’, ‘wearing glasses at night’, and ‘not wearing glasses at night’ [41]. Each acquisition situation includes a drowsy state, eye state, head state, and mouth state. Table 2 is organized by the detailed labels provided by the public DB NTHU-DDD.

NTHU-DDD DB is reconstructed into four classes of driver states using NTHU-DDD DB detailed labels for detailed classification of driver states. Driver state reconstruction organizes images inthe to normal class and drowsy class based on the drowsiness of NTHU-DDD. The DB composed of the normal class and drowsy class separates images where the subject’s mouth state is labeled as yawning to organize the yawning class. The DB composed of the normal class, drowsy class, and yawning class separates nodding and looking aside and talking and laughing label images from the normal class to organize the inattention class. Driver state labeling is organized by separating the driver’s drowsiness and distraction states. After state labeling, the driver states are defined as the drowsy class, normal class, yawning class, and inattention class.

Table 3 shows the information of StateFarm DB [42] and YawDD DB [43] used to compare the performance of driver inattention state recognition with NTHU-DDD DB through STFTransNet. StateFarm DB is a public DB of Kaggle used for classifying the state of driver concentration decline. StateFarm is a DB obtained from subjects of various races in an actual driving environment and consists of a normal driving state, a state of using a phone (one hand and two hands), a state of holding an object in the hand, a state of operating radio, a state of touching face or head, a state of drinking a beverage, a state of looking to the side or rear, and a state of operating a mobile phone on the lap (one hand or two hands). YawDD DB is a public DB used for classifying the state of driver yawning. YawDD is a DB obtained from subjects of various races in an actual driving environment and consists of a normal state, yawning state, and state of speaking or smiling.

The evaluation of the driver inattention state recognition system based on STFTransNet proposed in this paper was verified by the accuracy, which is the correct classification rate, and the F1 score, which is the harmonic mean of recall and precision, as shown in Equations (8–11). Table 4 shows the hyperparameter information of STFTransNet’s parameter set used for driver inattention state recognition. In this study, to evaluate the driver inattention state recognition system, we randomly shuffled the public DB’s NTHU-DDD, YawDD, and StateFarm to set the training, validation, and test data ratios to 6.5:1.5:2.

(8) $$Accuracy=\frac{TP+TN}{TP+FP+FN+TN}$$

(9) $$Precision=\frac{TP}{TP+FP}$$

(10) $$Recall=\frac{TP}{TP+FN}$$

(11) $$F1\;score=\frac{2\cdot Precision\cdot Recall}{Precision+Recall}$$

**Figure 7:**
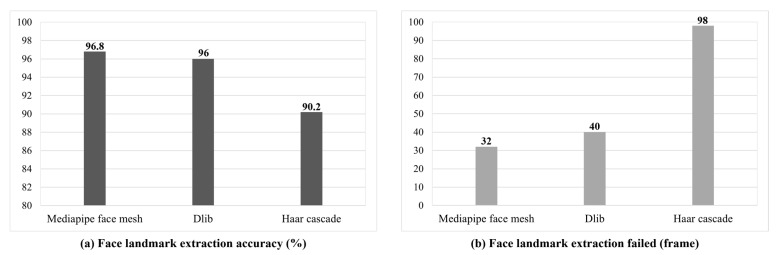
Comparison of feature extraction methods: (**a**) extraction accuracy and (**b**) failed extractions (count out of 1000 images).

Figure 7 is a graph comparing the accuracy and failure rate of facial features among mediapipe face mesh, Dlib, and Haar cascade, which are facial feature extraction techniques used for preprocessing the NTHU-DDD DB. Dlib extracts 68 facial feature points using a histogram of oriented gradients (HOG) and CNN [44]. Haar cascade is a classic facial feature extraction technique that detects faces based on Haar features [45]. As a result of the experiment, face extraction accuracy was 96.8% for mediapipe face mesh, 96% for Dlib, and 90.2% for Haar cascade, with mediapipe face mesh being the best. The number of failed recognition images among the three facial feature extraction methods was 32 for mediapipe face mesh, 40 for Dlib, and 98 for Haar cascade out of 1000 images. This study trained the proposed STFTransNet using only data from successful facial landmark detection. Since STFTransNet does not use data from failed facial landmark detections for model training, data from failed detections cannot affect the model.

Table 5 shows the accuracy of the proposed STFTransNet for driver inattention state recognition by comparing the frame intervals in the NTHU-DDD DB. The frames set used for analyzing the real-time driver inattention state recognition performance through the NTHU-DDD DB includes 10 frames, 15 frames, 20 frames, and 30 frames. As a result of the experiment, the driver inattention state recognition performance in 10 frames was the best, with an accuracy of 95.86% and an F1-score of 0.957. The proposed STFTransNet consumes 7.297 GFLOPs per frame. While the overall frame rate is 219 GFLOPs/s at 30 fps, 10-frame sampling reduces the real-time computational load to 21.9 GFLOPs/s, reducing the total processing load by approximately 10×. The 10-frame sampling method reduces memory overhead proportionally with the reduction in the number of frames processed, thereby enhancing stability in real-time driving environments.

**Figure 8:**
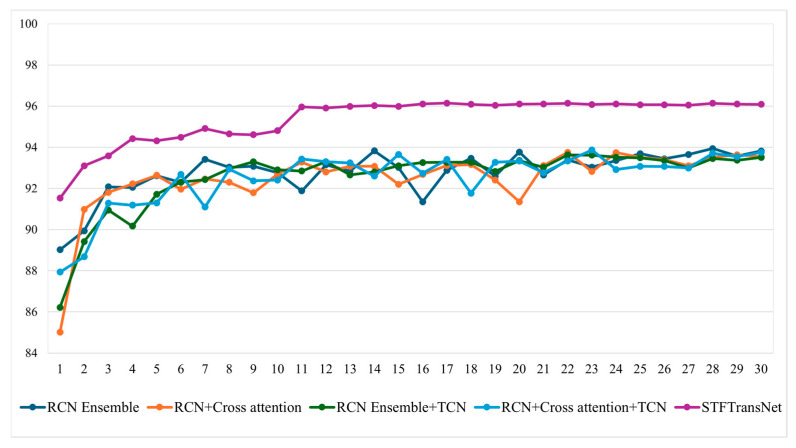
Stepwise network performance comparison chart.

Table 6 and Figure 8 show the performance change according to the step-by-step component combination process of the proposed STFTransNet. In experiments on block models, the proposed STFTransNet demonstrated that the two-stream RCN with cross-attention achieved 0.24% higher driver inattention recognition accuracy than the concatenation method. The concatenation method decreased driver inattention recognition accuracy by 0.31% when temporal features were extracted using TCN, while the cross-attention method improved accuracy by 0.11% when temporal features were extracted using TCN. The cross-attention method was selected as a suitable method for driver inattention recognition because it demonstrated higher driver inattention recognition accuracy and improved TCN learning performance compared to the concatenation method. Finally, the proposed STFTransNet achieved the best performance, with an accuracy of 95.86% and an F1-score of 0.957.

**Figure 9:**
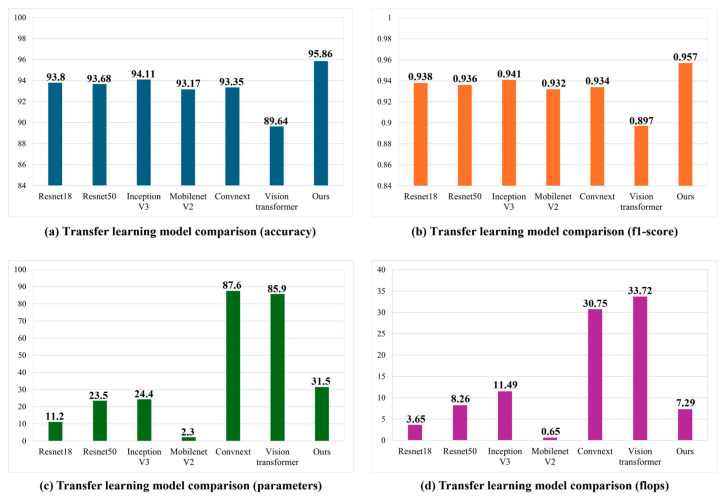
Performance comparison between proposed STFTransNet and transfer learning model: (**a**) accuracy, (**b**) F1-score, (**c**) parameters (M), and (**d**) FLOPs (per frame).

Figure 9 shows the performance comparison of the proposed STFTransNet and transfer learning models for driver inattention state recognition. InceptionV3 outperforms Resnet18 in driver inattention state recognition performance by 0.31% in accuracy and 0.003 in F1-score, but has approximately 2.18 times more parameters and 3.15 times more FLOPs. MobileNet V2 has 5.02 times fewer parameters and 5.59 times fewer FLOPs than Resnet18, making it suitable for real-time applications. However, its driver inattention state recognition performance is 0.63% lower in accuracy and 0.006 in F1-score. Resnet18 was selected as the backbone network due to its proven superior state recognition performance, low parameters, and FLOPs, complementing both MobileNet V2 and Inception V3. The proposed STFTransNet was compared with transfer learning-based models in terms of driver inattention state recognition accuracy, F1-score, parameters, and FLOPs and was found to have 1.75% greater accuracy, 0.016 higher F1-score, and 4.197 lower FLOPs than InceptionV3, demonstrating superiority in both driver inattention state recognition performance and real-time inference speed.

Table 7 shows the information comparing and analyzing the performance of the proposed STFTransNet with the existing driver inattention state recognition system using the NTHU-DDD DB. The existing driver inattention state recognition research developed a system that only recognizes drowsiness detection using the NTHU-DDD DB. The proposed STFTransNet recognizes four states, consisting of drowsy, normal, yawn, and inattention, to recognize both drowsiness and driver distraction. The proposed STFTransNet achieved an accuracy of 95.86% and an F1-score of 0.957, which is 14.86% at the maximum and 1.55% at the minimum, and an F1-score of 0.167 at the maximum and 0.076 at the minimum, which is superior to the existing models.

Table 8 shows information comparing the performance of the proposed STFTransNet with existing studies on the StateFarm DB. In the StateFarm DB, STFTransNet achieved 99.65% driver inattention state recognition accuracy and an F1-score of 0.996, which is 5.36% and 3.42% higher than the existing models.

Table 9 shows the information on the performance comparison analysis between existing driver inattention state recognition models and STFTransNet in the YawDD DB. In the YawDD DB, STFTransNet achieved an accuracy of 98.98% and an F1-score of 0.99, which is 6.88% higher and 0.33% higher than existing models, and an F1-score of 0.095 and 0.006, respectively.

Table 10 shows an analysis of information on Params, GFLOPs, Latency, Throughput, and Peak Memory of the proposed STFTransNet. The proposed STFTransNet model size is 31.53 M and requires 7.297 GFLOPs of computation per input frame. STFTransNet’s inference latency is measured at 0.176 ms per input frame, its throughput is 5678.9 frames per second, and its peak VRAM is 12.65 GB, including model weights, activations, and internal workspaces. The experimental data used in NTHU-DDD and YawDD were trained and tested by sharing training and testing data from the subjects, but not using them in duplicate. In StateFarm, the training and testing data were configured separately. The proposed STFTransNet achieved 3.48% higher accuracy than InceptionV3 + HRNN [12] in the separately configured StateFarm, and 4.56%, 3.78%, 3.16%, and 5.58% higher accuracy than VBFLLFA [11] and 2s-STGCN [15] in the shared configurations of NTHU-DDD and YawDD, respectively.

**Figure 10:**
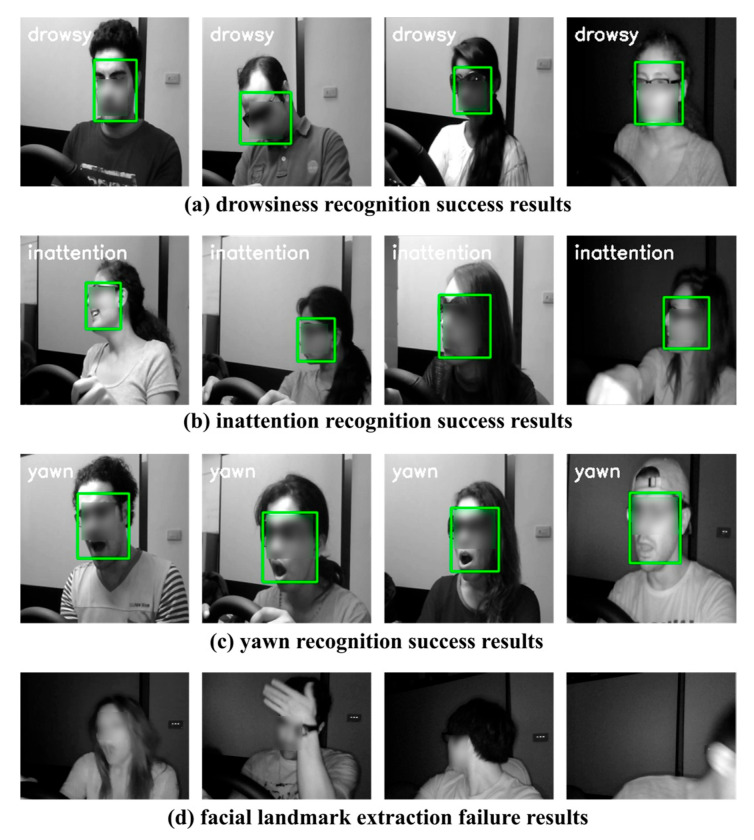
Results of a driver inattention recognition test using NTHU-DDD: (**a**) drowsiness, (**b**) inattention, (**c**) yawn, and (**d**) facial landmark extraction failure cases.

Figure 10 shows the result of driver inattention state recognition using NTHU-DDD. Figure 10a shows drowsiness recognition based on changes in the driver’s eye position and head angle. Figure 10b shows inattention recognition by detecting changes in the driver’s facial expression and head angle. Figure 10c shows the detection of the changes in the driver’s mouth shape and recognizes the yawn state. Figure 10d shows a case where facial landmark extraction fails. The proposed STFTransNet recognizes the driver’s normal state, drowsy state, inattention state, and yawn state in a situation where the face is occluded due to wearing sunglasses and glasses through multi-features of the driver’s body actions and face, as well as multi-dimensional feature extraction in spatial and temporal domains. Images with extreme facial occlusion or no driver within the camera frame cannot be used as experimental images for STFTransNet due to failed facial landmark detection. Failure to detect facial landmarks in real-world driving environments can lead to tracking gaps in driver inattention detection. Future studies are needed to address this issue of tracking gaps in driver inattention detection caused by failed facial landmark detection.

## 5. Conclusions

Studies are actively being conducted on driver inattention state recognition as an advanced mobility application technology to prevent traffic accidents caused by driver drowsiness and distraction. Driver inattention state recognition systems are typically divided into driver drowsiness recognition systems and driver distraction recognition systems, and they utilize vehicle operation data, driver driving behavior characteristics, and biosignals. Existing driver drowsiness recognition systems have difficulty improving driver inattention state recognition performance because of partial occlusion of the driver’s face image due to accessories worn on the face and light blurring caused by changes in the vehicle’s interior and exterior lighting, resulting in reduced momentary image resolution.

In this paper, we propose STFTransNet using facial features and body action features to solve the problem of state recognition failure due to feature information loss needed for state recognition. The proposed STFTransNet consists of (i) a mediapipe face mesh-based facial landmark extraction process for facial feature extraction, (ii) an RCN-based two-stream cross-attention process for learning spatial features of driver face and body action images, (iii) a TCN-based temporal feature extraction process for learning temporal features between extracted features, and (iv) an ensemble of spatial and temporal features and a classification process. As a result of the experiment, the proposed STFTransNet model for driver inattention state recognition system achieves 4.56% better accuracy than the existing VBFLLFA model in NTHU-DDD public DB, 3.42% better accuracy than the existing InceptionV3 + HRNN model in StateFarm public DB, and 3.78% better accuracy than the existing VBFLLFA model in YawDD public DB. The proposed STFTransNet has contributed to the development of improved system performance in recognizing driver drowsiness and distraction states in images with reduced resolution due to partial occlusion and momentary light scattering in driver face images. Future studies plan to build a database that includes drowsiness and distraction, and generalize the driver inattention state recognition system to occlusion and changing lighting situations by utilizing sophisticated face detection and a 3D-based feature extraction approach [48].

## Figures and Tables

**Table 1:** Driver inattention state recognition system technology trends.

Author	Data type	Acquisition data	Dataset	Network	Detect state	Acc.
McDonald et al. [16]	Vehicle	Steering wheel	Self-DB	RF	DDD	79%
Arefnezhad et al. [17]	Vehicle	Steering wheel	BI301Semi	ANFIS	DDD	98.12%
Huang et al. [10]	Behavior	Face/Hand	StateFarm	AWGRD	DID	95.97%
Ghourabi et al. [18]	Behavior	EAR, MAR	NTHU-DDD	MLP/K-NN	DDD	89.42%
Xiao et al. [19]	Behavior	Face	HNUFD	FPIRST	DDD	96.51%
Huang et al. [20]	Behavior	Face	NTHU-DDD	SMGA-Net	DDD	81%
Alotaibi et al. [12]	Behavior	Face/Hand	StateFarm AUC	InceptionV3 + HRNN	DID	96.23% 92.36%
Chaabene et al. [6]	Physiological	EEG	Self-DB	CNN	DDD	97.8%
Gangadharan et al. [21]	Physiological	EEG	Self-DB	SVM	DDD	78.3%
Shahbakhti et al. [22]	Physiological	EEG	acquisition DB [23,24,25]	VME-PCA-DWT	DDD	93%, 92%, 72.1%
Arefnezhad et al. [26]	Multi-modal	Steering wheel/ECG	Self-DB	KNN/RF	DDD	91.2%
Abbas et al. [27]	Multi-modal	PERCLOS/ECG	CAVE-DB/ DROWZY/ CEW	HybridFatigue	DDD	94.5%
Gwak et al. [28]	Multi-modal	Steering wheel/Face/EEG/ECG	Self-DB	RF	DDD	82.4%

**Table 2:** Detailed labels for NTHU-DDD.

Annotation	0	1	2
Fatigue	normal	fatigue	-
Eye	normal	sleep eyes	-
Head	normal	nodding	looking aside
Mouth	normal	yawning	talking and laughing

**Table 3:** Details regarding StateFarm and YawDD.

Detail	StateFarm	YawDD
subjects	26	21
Data type	Driver inattention	Driver Yawn
Number of samples	22,424	15,349
Class	10	3

**Table 4:** Experimental details.

Hyperparameters	Detail
Data split (Train:Validation:Test)	6.5:1.5:2
Batch size	64
Epoch	50
Learning rate	1 × 10^−4^
Weight decay	1 × 10^−3^
Activation function	GeLU
Optimizer	AdamW

**Table 5:** Comparison by data frame unit.

Frame	Best Acc. (%)	F1-Score
30	93.76	0.938
20	94.49	0.945
15	95.85	0.957
10	**95.86**	**0.957**

**Table 6:** Stepwise network performance comparison.

Model	Acc. (%)	F1-Score
RCN Ensemble	93.94	0.939
RCN + cross-attention	93.76	0.938
RCN Ensemble + TCN	93.63	0.936
RCN + cross-attention + TCN	93.87	0.938
**STFTransNet (Ours)**	**95.86**	**0.957**

**Table 7:** Comparative analysis of existing studies and proposed STFTransNet model using NTHU-DDD database.

Author	Method	Model	Acc. (%)	F1-Score	Class
Ghourabi et al. [18] (2020)	Eye closure	MLP/K-NN	94.31	0.790	2
Ed-Doughmi et al. [46] (2020)	Eye blinking	RNN	92.00	0.850	2
Bai et al. [15] (2022)	Face landmark	2s-STGCN	92.70	0.881	2
Yang et al. [11] (2024)	Face area	VBFLLFA	91.30	-	2
Huang et al. [20] (2024)	Face area	SMGA-Net	81.00	0.811	3
Ours	Driver area	STFTransNet	**95.86 ± 0.17**	**0.957 ± 0.002**	**4**

**Table 8:** Comparative analysis of existing studies and proposed STFTransNet model using StateFarm database.

Author	Method	Model	Acc. (%)	F1-Score
Abouelnaga et al. [14] (2018)	Face/Hand	AlexNet ensemble	94.29	-
Huang et al. [10] (2019)	Face/Hand	AWGRD	95.97	-
Alotaibi et al. [12] (2019)	Face/Hand	InceptionV3 + HRNN	96.23	-
Ours	Driver area	STFTransNet	**99.65 ± 0.13**	**0.996 ± 0.001**

**Table 9:** Comparative analysis of existing studies and proposed STFTransNet model using YawDD database.

Author	Method	Model	Acc. (%)	F1-Score
Mou et al. [47] (2021)	Eyes, Mouth, Head Flow	IsoSSL-MoCo	98.65	0.984
Yang et al. [14] (2021)	Face	3D CNN + BiLSTM	92.10	-
Bai et al. [15] (2022)	Face landmark	2s-STGCN	93.40	0.895
Yang et al. [11] (2024)	Face area	VBFLLFA	95.2	-
Ours	Driver area	STFTransNet	**98.98 ± 0.19**	**0.99 ± 0.002**

**Table 10:** Complexity and performance metrics of STFTransNet.

Metric	Unit/Condition	Value
Model size	Params (M)	31.53
GFLOPs	per frame	7.297
Inference Latency	per frame (ms)	0.176
Throughput	frames/s	5678.9
Memory	Peak VRAM(GB)	12.65

## Data Availability

The data provided in this study are available from the corresponding author.
